# Characterization of the complete chloroplast genome of the Eastern gamagrass, *Tripsacum dactyloides*

**DOI:** 10.1080/23802359.2017.1413312

**Published:** 2017-12-07

**Authors:** Yanfang Wang, Mingzhu Zhao, Li Li, Kangyu Wang

**Affiliations:** a College of Traditional Chinese Medicine, Jilin Agricultural University, Changchun, China;; b College of Life Science, Jilin Agricultural University, Changchun, China

**Keywords:** *Tripsacum dactyloides*, chloroplast genome, illumina sequencing, phylogenetic analysis

## Abstract

*Tripsacum dactyloides*, known as eastern gamagrass, is used as a donor of valuable traits. It grows naturally in the same region where maize is commercially cultured in the USA and has the ability to hybridize to maize. The wild genotype of eastern gamagrass is threaten by the gene flow from the transgenic maize. The circular genome is 141,050 bp in length and contains 120 genes, including 73 protein-coding genes (PCG), 39 transfer RNA genes (tRNA) and eight ribosomal RNA genes (rRNA). The overall nucleotide composition is: 30.8% A, 19.2% C, 19.3% G, 30.7% T, with a total G + C content of 38.5%. The phylogenetic tree was constructed to explore the taxonomic status of *Tripsacum dactyloides*, which contributes to phylogenetic studies and further conservation strategies for this species.

*Tripsacum dactyloides*, known as eastern gamagrass, belongs to the same tribe of the Poaceae family as maize (Zea mays L.) and is native to the United States. It is a donor of valuable traits such as drought tolerance (Berthaud and Savidan 1989), resistance to some diseases (Bergquist 1981), etc. Eastern gamagrass grows naturally in the same region where maize is commercially cultured in the USA and has the ability to hybridize to maize (Tsanev et al. 2002). In the USA, 70.9 million hectares were planted with transgenic corps in 2015 with high adoption rates for maize (92%) (James 2015). The gene flow from transgenic corps to their wild relatives might result in a decreased biodiversity and disruption of ecological equilibrium (Ellstrand 2001; Gewin 2003). Additionally, it will also cause the extinction of the wild genotype. Although no evidence exists of gene flow from maize to eastern gamagrass in nature, it is still necessary to protect the genetic resources of wild eastern gamagrass. In the present study, we obtained the complete chloroplast of wild eastern gamagrass, which will contribute to the conservation of this valuable genetic resource.

The specimen of *T. dactyloides* was isolated from Jilin Agricultural University test field in Changchun, Jilin, China (125.24E; 43.48N) and the DNA of *T. dactyloides* was stored in Jilin Agricultural University College of Life Science (No. JLAUCLS4). The sequencing step was performed on the Illumina X-Ten Sequencing Platform (Illumina, CA). Adapters and low-quality sequences were removed using FastQC software (Andrews 2010). The chloroplast genome was assembled with SPAdes v3.8 (http://bioinf.spbau.ru/spades) (Bankevich et al. 2012) and annotated by DOGMA (http://dogma.ccbb.utexas.edu/) (Wyman et al. 2004). The tRNA genes were further identified using ARAGORN (Laslett and Canback 2004). The annotated chloroplast genome was submitted to GenBank database under accession No.MG386499.

The chloroplast genome of eastern gamagrass was completed as a circular molecule of which the length is 141,050bp. It contained a pair of inverted repeat regions (IRs) of 22,750bp, a large single-copy region (LSC) of 82,955bp and a small single-copy region (SSC) of 12,555bp. Among these regions, a total of 120 genes were encoded, including 73 protein-coding genes (PCG), 39 transfer RNA genes (tRNA) and 8 ribosomal RNA genes (rRNA). In the IR region, 22 genes were found duplicated. Additionally, 17 genes were found containing a single intron. The base compositions of *M. spicata* chloroplast genome were uneven (30.8% A, 19.2% C, 19.3% G, 30.7% T). The overall GC content of this chloroplast genome was 38. 5%.

To ascertain phylogenetic position of eastern gamagrass among other higher plants, we selected 57 published complete chloroplast genome sequences of higher plants to construct alignment using Homblocks (https://github.com/fenghen360/HomBlocks) (Bi et al. 2017). The phylogenetic trees were reconstructed using maximum-likelihood (ML) and neighbour-joining (NJ) methods. ML analysis were performed using RaxML-8.2.4 (Stamatakis 2014), of which the bootstrap values were calculated using 1000 replicates to assess node support. NJ phylogenetic tree was constructed using MEGA7 with 5000 bootstrap replicate (Kumar et al. 2016). All the nodes were inferred with strong support by the ML and NJ methods. As shown in the phylogenetic tree (Figure 1), The chloroplast genome of eastern gamagrass was clustered with four plants in *Zea* genus.

**Figure 1.**
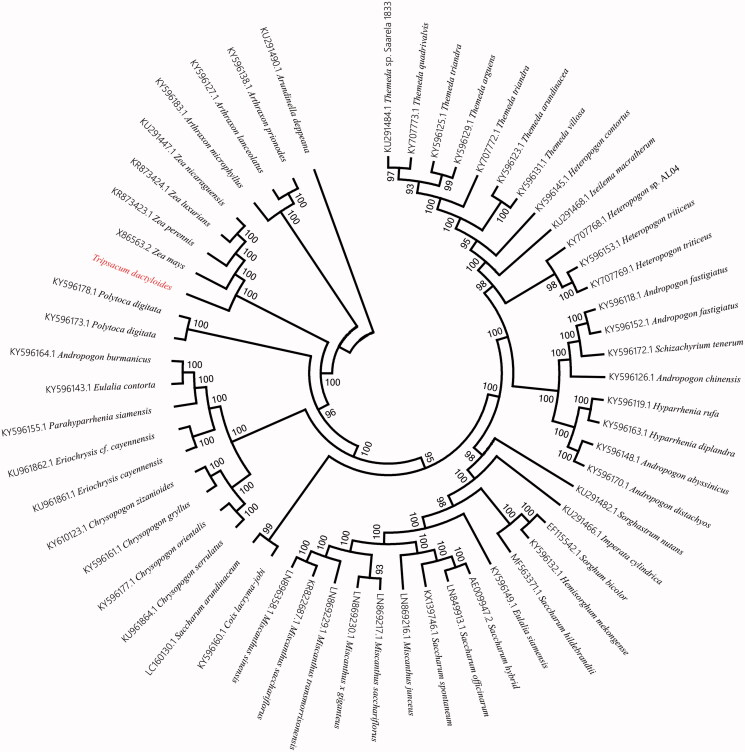
Phylogenetic relationships among 57 plant chloroplast genomes. This tree was drawn without setting of an outgroup. All nodes exhibit above 90% bootstraps. The length of branch represents the divergence distance.
